# First Assessment of Genetic Diversity, Phylogeographic Relationships, and Population Structure of the Brown Seaweed *Ericaria amentacea* From Italian Coasts Using Cytochrome Oxidase Subunit I (COI) Gene

**DOI:** 10.1002/ece3.74023

**Published:** 2026-07-13

**Authors:** Maha Moussa, Sarra Choulak, Valentina Asnaghi, Daniele Grech, Gareth Anthony Pearson, Khaled Said, Mariachiara Chiantore, Sonia Scarfi

**Affiliations:** ^1^ Department of Earth, Environment and Life Sciences (DISTAV) University of Genoa Genoa Italy; ^2^ National Biodiversity Future Center (NBFC) Palermo Italy; ^3^ Laboratory of Genetics, Biodiversity and Bioresources Valorisation (LR11ES41), Higher Institute of Biotechnology of Monastir University of Monastir Monastir Tunisia; ^4^ International Marine Centre (IMC), Loc. Sa Mardini Torregrande Oristano Italy; ^5^ Centre of Marine Sciences Universidade do Algarve Faro Portugal; ^6^ Inter‐University Center for the Promotion of the 3Rs Principles in Teaching & Research (Centro 3R) Torino Italy

**Keywords:** demographic expansion, distribution patterns, *Ericaria amentacea*, genetic structure, haplotype network

## Abstract

*Ericaria amentacea* is an endemic key seaweed species in the coastal ecosystem of the Mediterranean basin. Given its ecological role and sensitivity to environmental conditions decline, it has been designated as a protected species. Scarce local population genetic studies have been performed on this species on Italian coasts. For the first time, mitochondrial cytochrome oxidase subunit I (COI‐5P) gene was amplified and analyzed for 42 italian *E. amentacea* specimens, collected from five localities along Ligurian and Sardinian coasts. In addition, nine COI‐5P sequences were retrieved from National Center for Biotechnology Information (NCBI) investigating South Italy coasts. Polymorphism results revealed high values of haplotype diversity (*H*d) and very low nucleotide diversity (*π*). Thus, these results suggest that our *E. amentacea* populations may have undergone a genetic breakdown followed by rapid demographic expansion. This inference is strongly confirmed by the results of neutrality tests and “mismatch distribution”. The important number of haplotypes between localities and the high genetic differentiation (*F*st = 0.79) of the current *E*. *amentacea* populations could be maintained by the limited gene flow *Nm* (0.48). Moreover, results indicated that genetic variation is high, with most of it distributed among populations (79.39%). Both haplotype Network and biogeographic analysis showed a structured distribution according to the geographic origin. *E. amentacea* populations are subdivided into mainland versus insular populations. Indeed, these findings have important implications for conservation and restoration actions to counteract the potential decline of *E. amentacea* populations that require a deep knowledge of their genetic structure.

## Graphical abstract

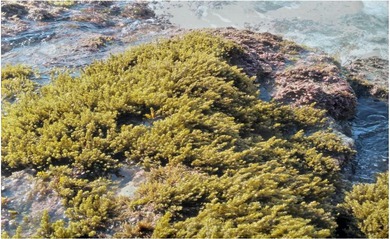

## 1 Introduction

The present‐day genetic diversity and phylogeographic structure of Mediterranean marine species have been shaped by a complex geological and climatic history that has strongly influenced connectivity patterns across the basin. During the Oligocene–Miocene, the counterclockwise rotation of the Corsica–Sardinia microplate and the opening of the Liguro–Provençal Basin contributed to the reorganization of the western Mediterranean and the isolation of Sardinia from the northwestern mainland (Schettino and Turco 2011; Advokaat et al. 2014), while subsequent tectonic processes, including the opening of the Tyrrhenian Sea and the uplift of the Apennines, further structured the central Mediterranean. The Messinian Salinity Crisis (5.96–5.33 Ma) caused extreme salinity fluctuations and major habitat loss, likely leading to strong demographic bottlenecks in marine biota (Krijgsman et al. 1999, 2024), whereas Quaternary glacial–interglacial cycles, particularly the Last Glacial Maximum, repeatedly altered sea levels and coastal habitats, promoting population isolation, refugial persistence, and postglacial recolonization (Lambeck et al. 2014; Patarnello et al. 2007; Maggs et al. 2008).

In addition, contemporary oceanographic and biogeographic barriers, such as the Strait of Messina and the Strait of Sicily, further contribute to genetic discontinuities due to their complex hydrodynamics and semi‐permeable connectivity (Villamor et al. 2014; Sanna et al. 2013). Together, these historical and present‐day processes have shaped the phylogeographic structure observed in Mediterranean marine species, including canopy‐forming brown macroalgae that play a key ecological role in temperate coastal ecosystems (Templado 2014; Thibaut et al. 2017).

**FIGURE 1:**
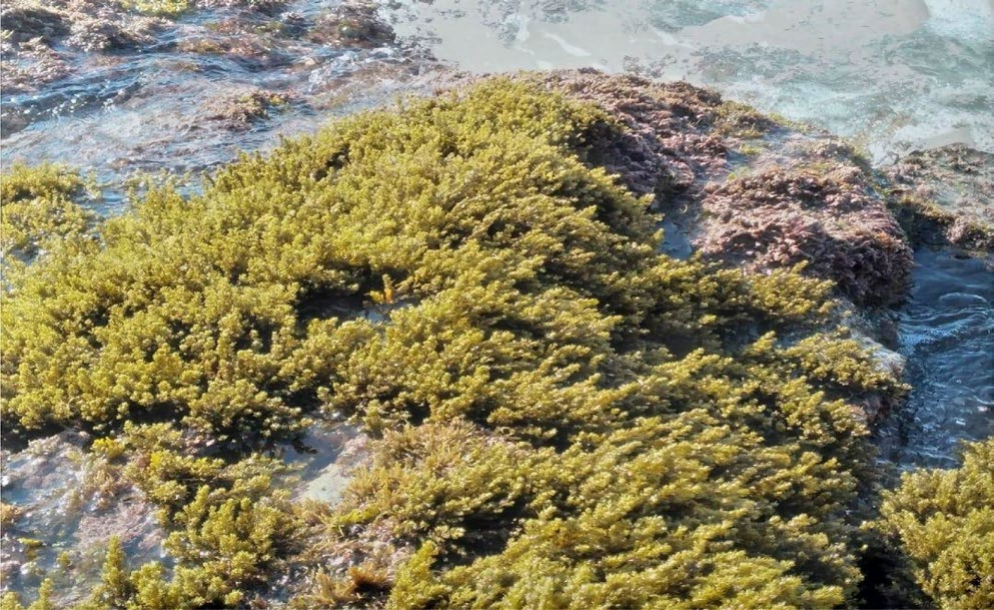
*Ericaria amentacea* forest in the intertidal zone along the Ligurian coast (photo by Maha Moussa in May 2024, Pontetto, Genova—Italy).

Among the species most sensitive to these historical and contemporary structuring processes are the canopy‐forming brown macroalgae, which are foundational components of temperate coastal ecosystems, providing structural habitat, influencing hydrodynamics, and supporting diverse communities of invertebrates and fishes (Steneck et al. 2002; Thibaut et al. 2015; Teagle et al. 2017; Wernberg et al. 2019; Bulleri et al. 2022). The decline of these macroalgal forests has been linked to coastal degradation, pollution, and climate change, resulting in loss of biodiversity and reduced ecosystem functionality (Sales et al. 2011; Mineur et al. 2015; Assis et al. 2017; Bevilacqua et al. 2019). Among these key species, *Ericaria amentacea* (C.Agardh) Molinari‐Novoa and Guiry, class Phaeophyceaeorder Fucales (Molinari‐Novoa and Guiry 2020; Figure 1), plays an essential ecological role along the Mediterranean coasts, including Italy, where it forms dense intertidal belts and acts as a habitat engineer (Falace et al. 2018; Mangialajo et al. 2008). Despite its importance, information about its genetic structure, population connectivity, and phylogeographic patterns remains scarce.

The taxonomy of the *Cystoseira C*. Agardh 1820 sensu lato complex, to which *E. amentacea* belongs, has been historically challenging due to pronounced morphological plasticity and frequent phenotypic overlap between species, often influenced by local environmental conditions (Bermejo et al. 2018; Orellana et al. 2019). This has complicated efforts to delineate populations and understand their phylogenetic relationships using morphology only. At the same time, increasing anthropogenic pressures such as coastal urbanization, eutrophication, and thermal stress are fragmenting populations and threatening their persistence (Thibaut et al. 2005; Susini et al. 2007). Assessing genetic differentiation or connectivity is therefore crucial to identify vulnerable populations, reconstruct dispersal pathways, and design effective conservation measures such as marine protected areas or restoration programs (Buonomo et al. 2017).

Previous molecular studies on *E. amentacea* have consistently revealed limited propagule dispersal, pronounced population genetic structure, and high sensitivity of local populations to habitat fragmentation and anthropogenic disturbances. Early work using RAPD markers in the Bay of Marseille (southern France) detected substantial genetic differentiation among nearby populations despite relatively high within‐population diversity, suggesting restricted effective dispersal of recruits (Susini et al. 2007). Subsequent analyses based on microsatellite (SSR) markers confirmed strong population structuring in the northwestern Mediterranean (Thibaut et al. 2016; Reynes et al. 2025) and along the southern Italian coast, where genetic differentiation has been associated with habitat discontinuity, coastal configuration, and stepping‐stone dispersal mediated by oceanographic currents (Buonomo et al. 2017). Collectively, these studies indicate that both biological traits and landscape features strongly influence connectivity and genetic structure in *E. amentacea*. However, these studies have largely been limited to RAPD and microsatellite markers. In contrast, the mitochondrial cytochrome c oxidase subunit I (COI‐5P) marker has become a widely used tool in marine organism research for assessing genetic diversity and phylogeography due to its high interspecific resolution, maternal inheritance, and extensive reference databases for marine organisms (Villamor et al. 2014; Sanna et al. 2013; Moussa et al. 2022). Regarding brown macroalgae, COI‐5P sequences have successfully revealed cryptic diversity within the *Cystoseira* sensu lato complex across the Mediterranean (Neiva et al. 2023). However, to date, no comprehensive COI‐based study has focused specifically on *E. amentacea* populations along the Italian coasts, leaving important gaps in our understanding of regional genetic differentiation and historical connectivity among major basins such as the Tyrrhenian, Adriatic, and Ionian Seas.

The present study provides the first assessment of genetic diversity, phylogeographic relationships, and population structure of *E. amentacea* along the Italian coasts using the mitochondrial COI‐5P gene. Through extensive sampling and molecular analyses, we aim to (1) describe the genetic diversity within and among Italian populations, (2) identify biogeographic barriers and potential historical glacial refugia or contact zones, and (3) assess levels of gene flow and connectivity relevant to conservation management. The outcomes will contribute to a baseline framework for monitoring genetic diversity and guiding conservation strategies for *E. amentacea* and other Mediterranean canopy‐forming brown algae.

## 2 Material and Methods

### 2.1 Sampling and Genomic DNA Extraction

**FIGURE 2:**
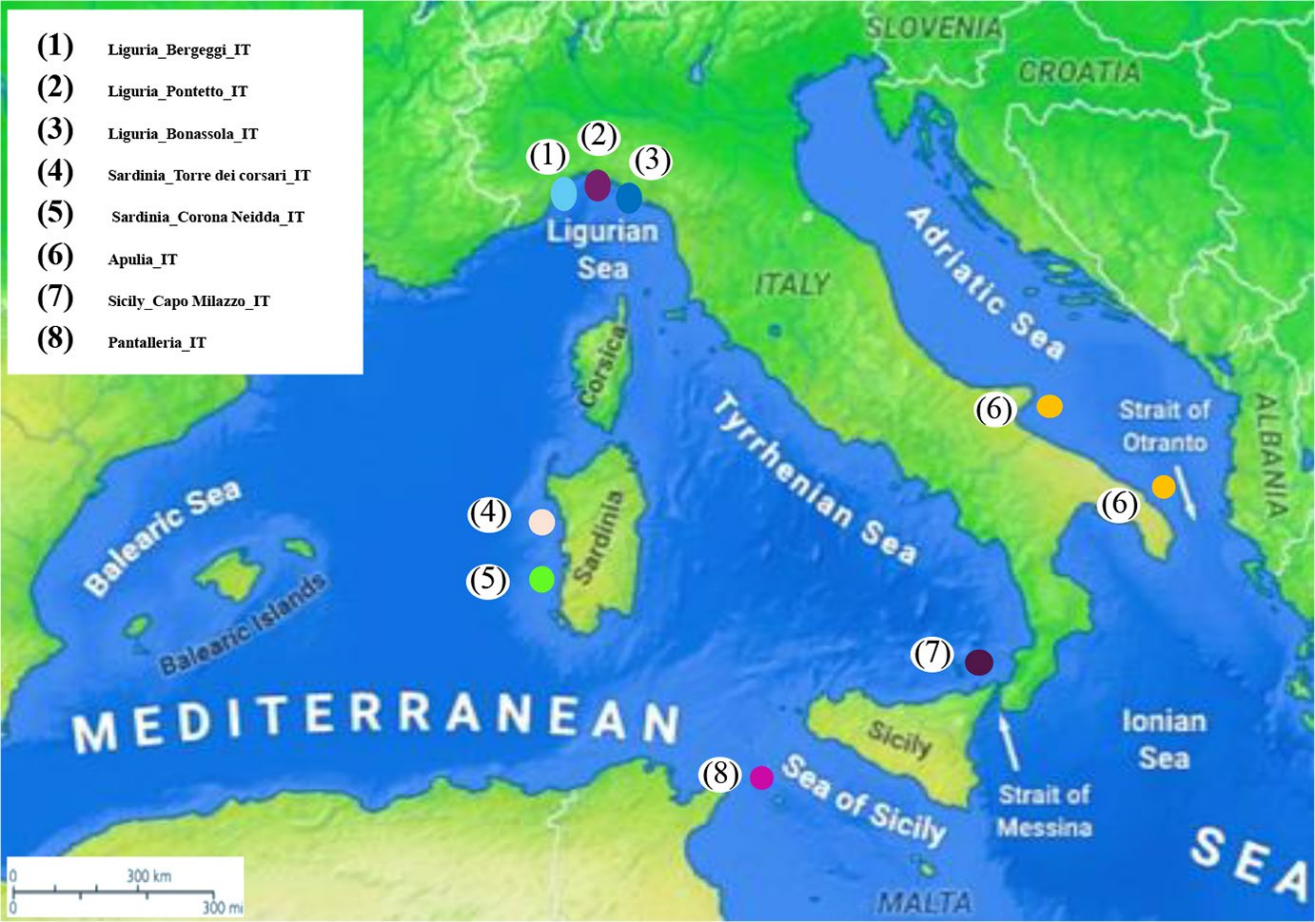
Geographical distribution of *E. amentacea* sampling.

A total of 42 specimens of *E. amentacea* were collected, between April and June 2023, from five sampling locations along the Italian coasts (Figure 2, Table 1). These samples covered the north board of Italy (Liguria region; Pontetto, Bergeggi and Bonassola) as well as Sardinia (Corona Niedda and Torre dei Corsari localities). From each specimen, a branch approximately 7–8 cm long was preserved in 100% ethanol and stored at 4°C for subsequent DNA extraction. Genomic DNA was extracted from 20 to 40 mg of the algal tissues using the CTAB method described by Zuccarello and Paul (2019), with some modifications adapted to the starting material. Samples were preserved in absolute ethanol and homogenized using a TissueLyser (Qiagen, Retsch) with stainless‐steel beads. Following chloroform extraction, samples were incubated at −80°C for 30 min during DNA precipitation to enhance DNA recovery. DNA quantity and quality evaluations were carried out using a NanoDrop One/One^c^ spectrophotometer (ThermoFisher, Milan, Italy) and agarose gel electrophoresis, respectively (Sambrook et al. 1989).

**TABLE 1:** Information on brown seaweed *E. amentacea* sampling including collection region, collection site, population size (N), site collection depth, and GPS coordinates.

Collection region	Collection site	N	Collection depth	GPS coordinates
Italian mainland	Liguria_Bergeggi (1)	10	Tide level from 0 to 20 cm	44°14′55″ N, 8°26′37″ E
	Liguria_Pontetto (2)	10	Tide level from 0 to 20 cm	44°22′33″ N, 9°4′31″ E
	Liguria_Bonassola (3)	8	Tide level from 0 to 20 cm	44°10′60″ N, 9°34′60″ E
	Apulia (6)	3	—	Data from NCBI
Italian Islands	Sardinia: *Torre dei Corsari (4) * Corona Niedda (5)	8 6	Tide level from 0 to 20 cm	39° 40′ 45.18″ N, 8° 27′ 05.06″ E 40° 12′ 45.47″ N, 8° 27′ 31.21″ E
	Pantelleria (8)	2	—	Data from NCBI
	Sicily_Capo Milazzo (7)	4	—	Data from NCBI

A set of *E. amentacea* COI‐5P sequences was retrieved from the National Center for Biotechnology Information (NCBI). These data covered the south of Italy (Sicily (Capo Milazzo) and Pantelleria islands and Apulia region).

### 2.2 Mitochondrial DNA Amplification and Sequencing

The mitochondrial fragment of COI‐5P was amplified using a pair of primers; COI‐Frwd: 5′‐CCAACCAYAAAGATATWGGTAC‐3′ and COI‐Rev: 5′‐GGATGACCAAARAACCAAAA‐3′ (Saunders and McDevit 2013). PCR reactions were performed in a total volume of 25 μL including 2 μL (20 ng/μL) of DNA, 2.5 μL PCR Buffer (10×), 3.2 μL MgCl_2_ (20 mM), 0.5 μL of each primer (10 μM), 0.25 μL dNTP mix (20 mM), 1.25 μL BSA (10 mg/mL), 0.25 μL (1 U/μL) of Taq DNA polymerase, and sterile double‐deionized H_2_O. PCR amplifications were performed in an Eppendorf AG Thermocycler, programmed to perform an initial denaturation at 94°C for 4 min; followed by 38 cycles at 94°C for 1 min, 50°C for 30 s, and 72°C for 1 min; and a final extension at 72°C for 7 min. PCR amplicons were screened for specific fragment size on 1.5% agarose gel electrophoresis and subsequently purified using a Gel and PCR Clean‐up (MACHEREY‐NAGEL, Germany) purification kit. The agarose gel was photographed by iBright 1500 imager (Invitrogen, Thermofisher).

Amplified PCR products were sequenced using the Sanger method (Sanger et al. 1977) at Eurofins Genomics Germany GmbH (Ebersberg, Germany) and aligned using MUSCLE (Edgar 2004) implemented in MEGA version 11.0 (Tamura et al. 2021).

### 2.3 Data and Statistical Analysis

#### 2.3.1 Analysis of Genetic Variability

Measures of genetic diversity as number of haplotypes (H), haplotype diversity (Hd; Nei 1987), and nucleotide diversity (π; Tajima 1983; Nei 1987) were estimated across the entire dataset using DnaSP version 5.10 (Librado and Rozas 2009). Theta (per site) from Eta, the average number of pairwise differences (K), transition/transversion bias (R), and the number of variable and parsimony‐informative nucleotide sites were calculated using MEGA version 11.0 (Tamura et al. 2021).

#### 2.3.2 Demographic History

Mismatch distribution analysis was performed using DnaSP software version 5.10 (Librado and Rozas 2009) for the COI‐5P dataset. To assess deviations from neutrality, demographic expansion, or the detection of selection signatures, additional tests were carried out based on the total number of mutations, including Tajima's D (Tajima 1989), Fu's Fs (Fu and Li 1993), the raggedness index (rg), and Ramos‐Onsins and Rozas's *R* ^2^ (Ramos‐Onsins and Rozas 2002). These analyses were performed using coalescent simulations implemented in DnaSP, with 1000 simulated resampling replicates.

#### 2.3.3 Population Structure Analyses

To infer the haplotype relationships of *E. amentacea* haplotypes, a haplotype network was constructed in PopART version 1.7 software (Leigh et al. 2015) using the median‐joining method (Bandelt et al. 1999). Phylogenetic hypotheses were built using the Neighbor‐Joining (NJ) method implemented in MEGA version 11.0 (Tamura et al. 2021). A sequence from *Ericaria zosteroides* (Turner) Molinari‐Novoa and Guiry 2020 (GenBank accession numbers: OK480329.1) was used as an outgroup.

#### 2.3.4 Genetic Differentiation Analyses

The analysis of molecular variance (AMOVA; Excoffier et al. 1992) was conducted using Arlequin version 3.5 (Excoffier and Lischer 2010) to assess the level of genetic differentiation among Italian populations of *E. amentacea*. Two supplementary AMOVA tests were performed. In the first analysis, genetic variation among populations was examined according to geographic proximity: Liguria_Bergeggi/Liguria_Bonassola/Liguria_Pontetto/Sardinia_Torre dei Corsari/Sardinia_Corona Niedda/Sicily_Capo Milazzo/Apulia/Pantelleria. The second analysis compared continental versus insular populations, grouped as follows: Liguria_Bergeggi/Liguria_Bonassola, Liguria_Pontetto/Apulia (continental) versus Sardinia_Torre dei Corsari/Sardinia_Corona Niedda/Sicily_Capo Milazzo/Pantelleria (insular). All AMOVA analyses were performed with 10,000 permutations under null distributions.

The extent of genetic differentiation among populations was further estimated using fixation indices *F*_ST_ (Wright 1931), GST (Nei 1975), and NST (Lynch and Crease 1990) as well as gene flow Nm (Hudson et al. 1992). These values were calculated with 1000 data permutations using DnaSP version 5.10 (Librado and Rozas 2009).

## 3 Results

### 3.1 Genetic Diversity

The *Ericaria amentacea* COI‐5P dataset, composed of 51 sequences, has a final alignment length of 610 bp, of which 585 sites were conserved and 25 were variable (Table 2). Among these variable positions, 14 were informative on parsimony and 11 were unique sites, corresponding to a total of 28 mutations detected. Overall, 25 polymorphic (variable) sites were identified across the aligned sequences, resulting in 17 distinct haplotypes among the studied populations. Haplotype diversity (Hd) was 0.851, and nucleotide diversity (Pi) was 0.00597. The mean number of pairwise nucleotide differences (K) between sequences was 3.64.

**TABLE 2:** Summary of polymorphism of COI sequences.

	COI sequences
Number of sequences	51
Alignment length (bp)	610
Conserved sites	585
Variable sites	25
Parsimony informative characters	14
Singleton variable sites	11
Total number of mutations	28
Number of polymorphic sites (S)	25
Number of haplotypes (H)	17
Haplotype diversity (Hd)	0.851
Variance of haplotype diversity	0.00165
Nucleotide diversity (Pi)	0.00597
Theta (per site) from Eta	0.01020
Average of pairwise differences (K)	3.64471
Transition/transversion bias (R)	1.06

### 3.2 Demographic History

The neutrality tests applied to the dataset produced predominantly negative values for different statistics (Table 3). Tajima's D was negative (*D* = −1.36603) but did not reach statistical significance (Tajima 1989). Likewise, D* (−1.89830) and F* (−2.03313) of Fu and Li were negative but not significant. Fu's Fs was strongly negative (*Fs* = −4.536).

**TABLE 3:** Tajima's *D*, Fu's *F*S, Ramos‐Onsins and Rozas's (*R*2), Fu and Li's D* and Fu and Li's F* *neutrality* tests and mismatch distribution raggedness index (*r*) for the entire *E. amentacea* samples.

Geographic group	*Tajima's D*	*Fu's Fs*	*R*2	*R*	Fu and Li's D*	Fu and Li's F*
All data set	−1.36603 ns	−4.536	0.0674	0.0275	−1.89830 ns	−2.03313 ns

Abbreviation: ns, nonsignificant.

Two demographic indices complemented these results (Table 3). The R^2^ statistic was low (*R* ^2^ = 0.0674) (Ramos‐Onsins and Rozas 2002), as was the raggedness index (*r* = 0.0275) (Harpending 1994).

**FIGURE 3:**
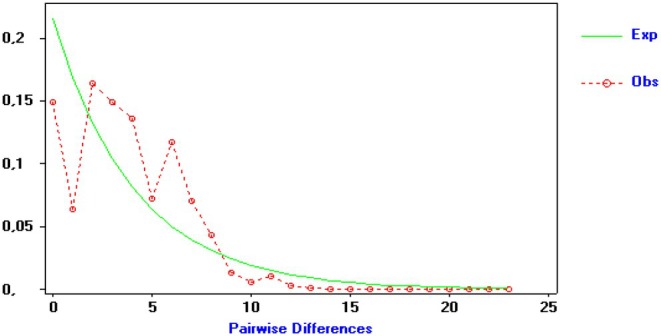
Mismatch distribution of pairwise nucleotide differences using DNAsp for *Ericaria amentacea* populations from Italian coasts.

The mismatch distribution for *Ericaria amentacea* populations from Italy showed a unimodal curve, with a single clear peak at low pairwise differences (Figure 3).

### 3.3 Genetic Differentiation Analyses

The analysis of molecular variance (AMOVA) highlighted a marked and notable genetic structure among the populations studied (Table 4).

**TABLE 4:** Molecular variance analysis (AMOVA) of *p < 0.05.

AMOVA: all dataset Liguria_Bergeggi vs. Liguria_Bonassola vs Liguria_Pontetto vs. Sardinia_Torre dei Corsari vs Sardinia_Corona Niedda vs. Sicily_Capo Milazzo vs. Apulia vs Pantelleria				
Source of variation	Fixation index	Sum of squares	Variance components	Pourcentage of variation
Among populations	*Φ*_ST_ = 0.79391	417.217	7.899	79.39085*
Within populations		88.175	2.051	20.60915*
Total		505.392	9.950	100
**AMOVA groups: continent vs islands** **Liguria_Bergeggi/Liguria_Bonassola/Liguria_Pontetto/Apulia vs Sardinia_Torre dei Corsari/Sardinia_Corona Niedda vs Sicily_Capo Milazzo vs Pantelleria**				
Among groups	*Φ*_SC_ = 0.60393 *Φ*_ST_ = 0.86105 *Φ*_CT_ = 0.64917	318.180	9.580	64.91716*
Among populations within groups		99.037	3.127	21.18764*
Within populations		88.175	2.051	13.89519*
Total		505.392	16.610	100

According to a global analysis, the majority of genetic diversity (79.39%) was distributed among *E. amentacea* populations, whereas only 20.61% was maintained within populations (Φ_ST_ = 0.79391, *p* < 0.05). This indicates a restricted gene flow and significant genetic differentiation among populations.

The division of the data set into continental and island populations highlighted an even more pronounced structuring (Table 3). Most of the genetic variation (64.92%) was due to disparities between the two groups, while 21.19% were due to the variation existing between populations within each group, and only 13.89% were preserved within populations (ΦCT = 0.64917; Φ_SC_ = 0.60393; Φ_ST_ = 0.86105; *p* < 0.05).

Pairwise estimates of genetic differentiation (FST) and gene flow (Nm) revealed a pronounced genetic structuring among populations (Table 5). Most comparisons showed very high levels of differentiation, with FST values ranging from 0.68 to 0.92. For instance, Sardinia_Corona Niedda and Sicily_Capo Milazzo were almost completely differentiated (FST = 0.92), and similarly high values were observed between Sardinian, Sicilian, and Pantelleria populations. In contrast, continental Ligurian populations displayed low levels of differentiation. Pairwise comparisons between Liguria_Pontetto and Liguria_Bonassola (*F*_ST_ = 0.04) and between Liguria_Pontetto and Liguria_Bergeggi (*F*_ST_ = 0.05) revealed low genetic structuring. The corresponding Nm values (4.87 and 4.37, respectively) indicated significant gene flow among close Ligurian populations.

**TABLE 5:** Pairwise comparisons of genetic differentiation, estimated from haplotype frequencies (*F*_*ST*_, above the diagonal) and gene flow (*N*_*m*_, below the diagonal).

	Pantelleria	Apulia	Liguria_Bergeggi	Sardinia_Corona Niedda	Sardinia_Torre dei Corsari	Sicily_Capo Milazzo	Liguria_Bonassola	Liguria_Pontetto
Pantelleria	0	1	0.81	1	0.88	1	0.86	0.81
Apulia	0	0	0.69	1	0.88	1	0.76	0.69
Liguria_Bergeggi	0.06	0.11	0	0.76	0.86	0.68	1 (−0.03)	0.05
Sardinia_Corona Niedda	0	0	0.08	0	0.88	1	0.72	0.76
Sardinia_Torre dei Corsari	0.05	0.03	0.06	0.03	0	0.92	0.84	0.80
Sicily_Capo Milazzo	0	0	0.11	0	0.02	0	0.76	0.69
Liguria_Bonassola	0.04	0.08	0 (−7.62)	0.05	0.05	0.08	0	0.04
Liguria_Pontetto	0.06	0.11	4.37	0.07	0.06	0.11	4.87	0

### 3.4 Population Structure Analyses

**FIGURE 4:**
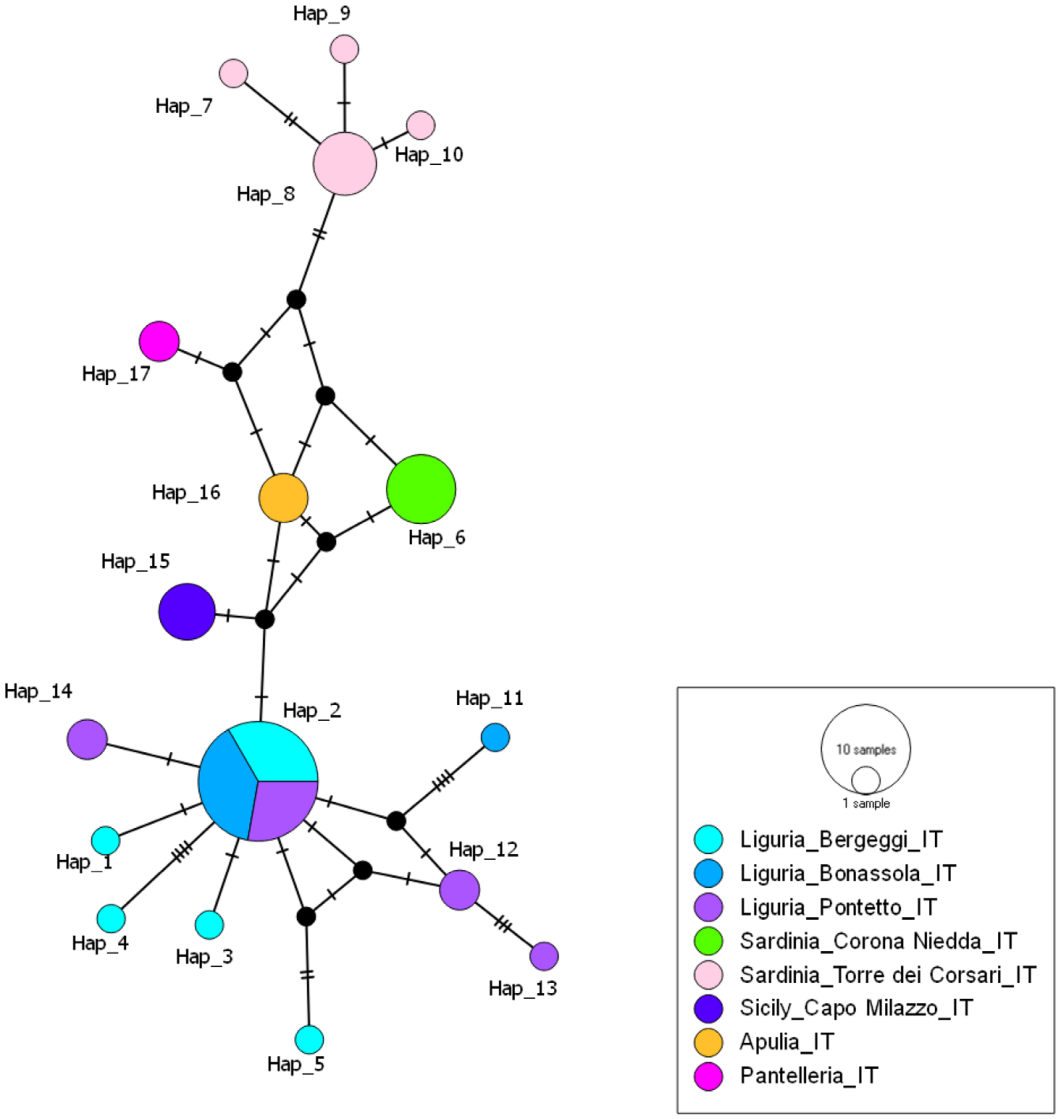
The median‐joining haplotype network constructed from COI sequences for *Ericaria amentacea* populations along Italian coasts.

Haplotype network analysis identified 17 haplotypes organized into six main haplogroups, interconnected through five inferred lost haplotypes (Figure 4).

The haplogroup I is the largest group, comprising 9 haplotypes (Hap1 to Hap5, Hap11 to Hap14) centered on Hap2, the most frequent and likely ancestral haplotype (*n* = 18). All haplotypes within this haplogroup are connected to Hap2 by one to four mutational steps.

The haplogroup II comprises 4 haplotypes (Hap7, Hap8, Hap9, Hap10), with Hap8 (*n* = 5) as the most frequent. This haplogroup is separated from Haplogroup I by 2 inferred haplotypes and a minimum of 4 mutational steps.

While haplogroup III is represented by a single haplotype, Hap6 (*n* = 6), connected to Haplogroup I via 1 inferred haplotype and 3 mutational steps.

**FIGURE 5:**
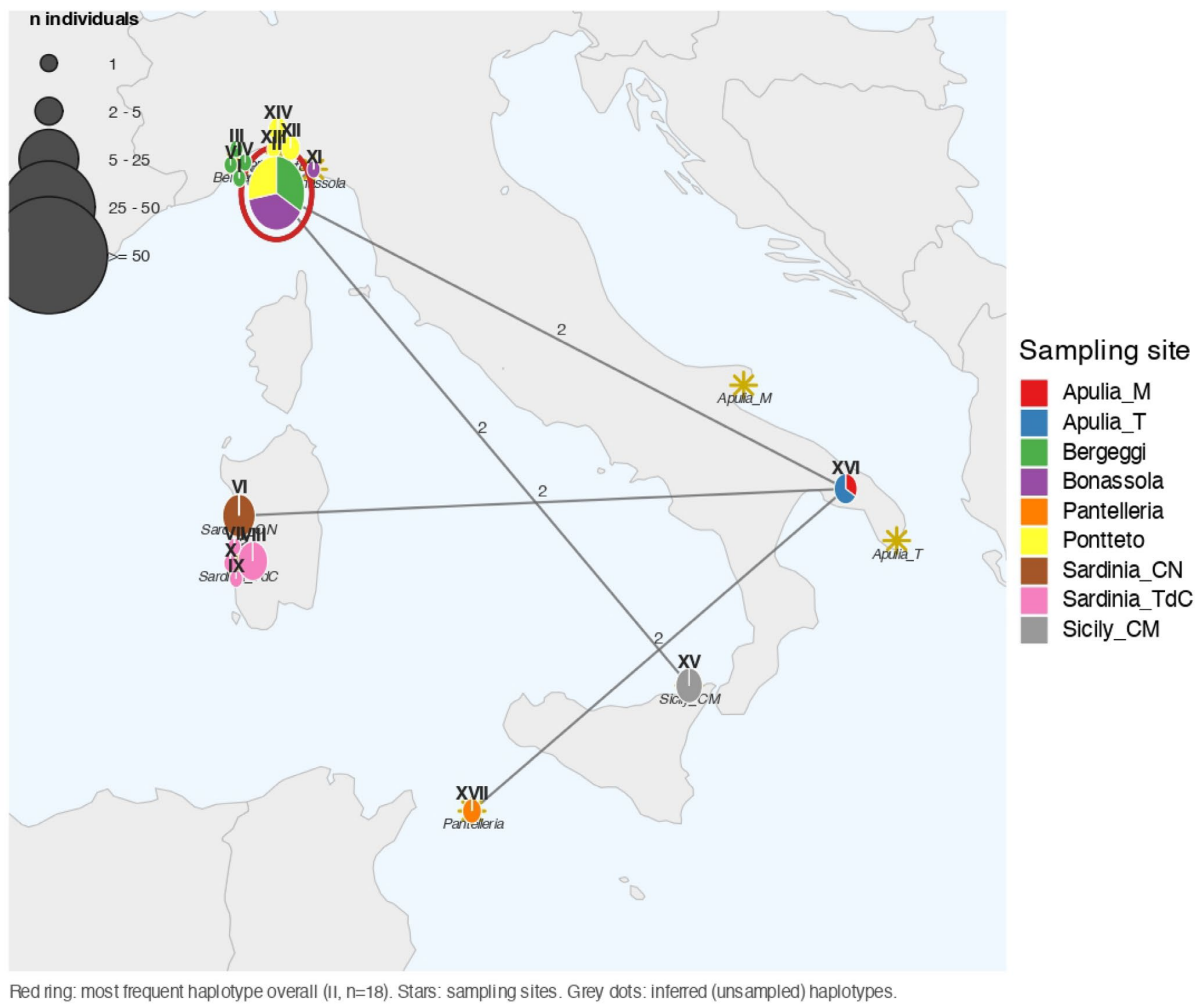
Geographic distribution of haplotypes identified in *Ericaria amentacea* populations from the Italian coasts.

Three additional haplotypes occupy peripheral positions in the network. Haplotype IV (Hap15; *n* = 4) is directly linked to Hap2 by a single mutational step. Haplotype V (Hap16; *n* = 3) is separated from the central cluster by two mutational steps through one inferred intermediate haplotype. Haplotype VI (Hap17; *n* = 2) is the most divergent, connected to the central cluster via two inferred haplotypes and the highest number of mutational steps. The haplotype distribution map (Figure 5) shows that Haplogroup I is exclusively concentrated along the northern Ligurian coast, where Bergeggi, Bonassola, and Pontetto share the dominant Hap2 alongside several private haplotypes, reflecting high local haplotype diversity. Sardinian populations are represented by two geographically distinct groups: Corona Niedda in the north, exclusively carrying Hap6 (Haplogroup III), and Torre dei Corsari in the west, exclusively carrying Hap7, Hap8, Hap9, and Hap10 (Haplogroup II). Sicily (Capo Milazzo) is exclusively represented by Hap15, while the two Apulian sites (Apulia_M and Apulia_T) carry Hap16 exclusively. Pantelleria, the southernmost and most geographically isolated site, is exclusively represented by Hap17. All peripheral populations (Sardinia, Sicily, Apulia, and Pantelleria) are connected to the Ligurian cluster by 2 mutational steps, and none of their haplotypes are shared with any other sampling site.

**FIGURE 6:**
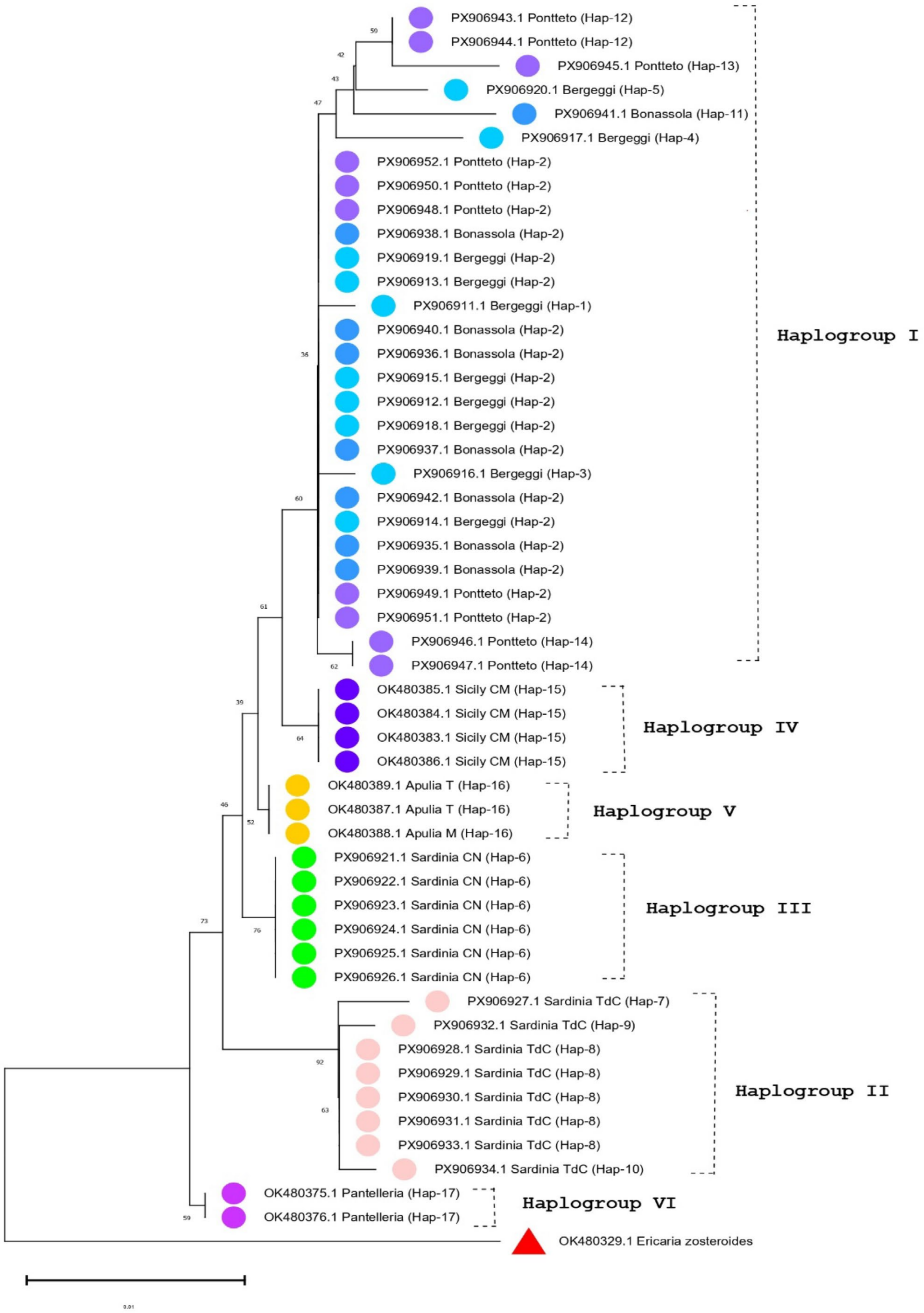
Neighbor‐Joining phylogenetic analysis of *E. amentacea* populations along the Italian coasts. Sardinia TDC: Sardinia Torre dei Corsari, Sardinia CN: Sardinia Corona Neidda, Apulia T: Apulia Tricase, Apulia M: Apulia Mattinata, Sicily CM: Sicily Capo Milazzo.

The Neighbor‐Joining phylogenetic analysis of *E. amentacea* populations along the Italian coasts revealed a distinct structure largely shaped by geographic location (Figure 6), supporting the phylogeographic divergence observed by the median‐joining haplotype network (Figure 4). With bootstrap values ranging from 47 to 62, samples from the Ligurian region (Pontetto, Bergeggi, and Bonassola) formed a substantially monophyletic group, suggesting a notable genetic consistency within northern populations. However, the samples from Apulia and Sicily (Capo Milazzo) showed significant genetic divergence, forming a distinct lineage, as reflected by intermediate bootstrap values (65%–52%). Two geographically separate sets of Sardinian populations have been identified: one that includes samples from Torre dei Corsari and the other that includes samples from Corona Niedda. The genetic differentiation between northern and southern Sardinian locations (bootstrap 73%) suggests limited connectivity and potential regional divergence.

Compared to all other populations, Pantelleria specimens, forming a special and unique clade at the base of the *E. amentacea* group, showed the highest level of genetic divergence. The Pantelleria population is very isolated, according to this model. The monophyly of *E. amentacea* was confirmed by the inclusion of *E. zosteroides* as an outgroup; all intraspecific variations revealed stronger ties between them than with this related species.

## 4 Discussion

Phylogeography studies of *Ericaria amentacea* are important for understanding its evolutionary and ecological dynamics. By investigating genetic variability within and among populations, researchers can uncover historical processes such as migration patterns, geographical isolation, and recolonization events that shaped their current distribution. These approaches also enable the identification of the discrete genetic lineages, emphasizing significant intraspecific variation, which is critical for resilience and adaptation to environmental changes, particularly under global warming and anthropogenic pressures on coastal ecosystems. Furthermore, phylogeography provides significant insights for population management and conservation by describing major evolutionary units and informing protection strategies to ensure the genetic diversity and stability of marine communities, where this species plays an important ecological function.

The analysis of genetic parameters is fundamental for understanding the structure, diversity, and evolution of natural populations. These parameters, such as haplotype diversity, nucleotide diversity, the number of polymorphisms, or even genetic differences between populations, allow the assessment of genetic diversity levels of a species, a key indicator of its adaptability to environmental changes and anthropogenic pressures.

Polymorphism results on *E. amentacea* populations revealed very low nucleotide diversity (Pi = 0.00597) and high haplotype diversity (Hd = 0.851), indicating a moderate to high intraspecific genetic variation. These results are consistent with expectations for a species with geographically structured populations that preserved multiple distinct lineages across its distribution range. The average number of pairwise differences (*K* = 3.64) suggests the presence of distinct haplotype lineages within populations, which could represent both historical isolation and limited contemporaneous gene flow (Rogers and Harpending 1992; Provan and Maggs 2011).

The combination of a strongly negative Fu's Fs, low *R* ^2^ and low raggedness values, together with the unimodal mismatch distribution, despite nonsignificant values for Tajima's D and Fu & Li's statistics, is generally interpreted as a signal of recent demographic expansion, often following a reduction in population size (bottleneck) and a rapid accumulation of rare alleles during recolonization (Rogers and Harpending 1992; Fu 1997). This pattern has already been reported in several marine macroalgae and other marine organisms and is consistent with postglacial scenarios of recolonization and rapid expansion along the Mediterranean coasts (Bermejo et al. 2018; Veith et al. 2020).

However, because these neutrality and demographic statistics were computed on the pooled dataset, they reflect an average signal that may not be representative of all populations equally. The haplotype network provides a more detailed picture of this demographic signal: the northern Ligurian populations (Bergeggi, Bonassola, Pontetto) cluster tightly around the most frequent haplotype (haplotype II, *n* = 18) and share several closely related haplotypes, consistent with high connectivity and a genuine recent expansion in this region. In contrast, peripheral populations (Sardinia, Sicily (Capo Milazzo), and Pantelleria) are characterized by highly divergent haplotypes separated from the main network by long mutational paths, and several of these populations showed no internal polymorphism, suggesting historical isolation and restricted contemporary gene flow (Provan and Maggs 2011). This contrast indicates that the demographic expansion signal detected at the pooled level is not uniform across the species' range, but is largely driven by the genetically diverse and well‐connected northern cluster. Such spatial heterogeneity is not consistent with a single, range‐wide bottleneck, which would be expected to leave a comparable signature across all populations; rather, it points to a scenario of genetic breakdown, in which a barrier to gene flow has progressively restricted connectivity between a diverse, expanding core and isolated peripheral populations that have retained little to no internal variation (Thibaut et al. 2016; Riquet et al. 2021).

These contrasting demographic trajectories among populations can be understood in the context of post‐glacial history. Major climate changes at the end of the last glaciation, approximately 18,000 to 10,000 years ago, profoundly transformed Mediterranean coastal habitats through decreasing temperatures and sea‐level fluctuations, leading to the reduction or fragmentation of rocky substrates favorable to macroalgae and other benthic organisms (Rohling et al. 1998; Patarnello et al. 2007; Lambeck et al. 2014). In this context, several temperate marine species are thought to have survived in glacial refugia located mainly in the southern and eastern regions of the Mediterranean, particularly along the North African coasts, Sicily, Sardinia, and Greece, where environmental conditions remained relatively stable (Maggs et al. 2008). Following the Last Glacial Maximum (LGM, approximately 23,000–19,000 years ago), the return of suitable conditions along northern coasts likely enabled rapid recolonization from these refugial sources, generating the expansion signal observed in Ligurian populations, while southern and insular populations retained the signature of longer‐term isolation.

This pattern is consistent with the population structure described below, where geographic and oceanographic barriers, such as the Strait of Messina, recognized as a major genetic discontinuity zone in Mediterranean marine taxa (Patarnello et al. 2007), as well as the isolation of Pantelleria and southern Sardinia, appear to have played a key role in shaping contrasting demographic trajectories among *E. amentacea* populations, allowing some lineages to expand while others remained demographically static or genetically depauperate.

The AMOVA results clearly highlight a strong genetic structuring within *E. amentacea* populations. Indeed, the majority of the total genetic variability (79.39%) is attributed to differences between populations (Φ_ST_ = 0.79391; *p* < 0.05), while a lower proportion (20.61%) is observed within populations. This distribution reflects restricted gene flow between populations and marked genetic differentiation, likely related to the low dispersal of propagules and the sedentary nature of spores and gametes in the *Cystoseira* group. Despite their high reproductive potential and the production of abundant zygotes, dispersal remains extremely limited because the relatively large zygotes (~100 μm) have a short planktonic lifespan and rapidly sink and attach to the substrate. Consequently, recruitment typically occurs in close proximity to parental individuals, often within a few centimeters of the parental thalli. This restricted dispersal reduces effective gene flow among populations and promotes local genetic structuring, contributing to the persistence of marked genetic differentiation even among geographically nearby populations (Mangialajo et al. 2012; Gianni et al. 2013). Furthermore, the observed genetic structure may be reinforced by priority effects, whereby established populations limit the successful establishment of incoming recruits, thereby reducing effective gene flow even when propagule dispersal occurs (De Meester et al. 2002; Neiva et al. 2012; Thibaut et al. 2016). The strong genetic differentiation observed in the present study is consistent with previous findings for *Ericaria amentacea* (formerly *Cystoseira amentacea*) across the Mediterranean. Significant population structuring has been reported in the Bay of Marseille (southern France) and northwestern Mediterranean coast (Susini et al. 2007; Thibaut et al. 2016; Reynes et al. 2025; using microsatellite and RAPD markers), where restricted connectivity was attributed to the limited dispersal capacity of propagules, habitat discontinuity, and oceanographic barriers. Similar patterns have also been documented in other members of the former *Cystoseira* sensu lato group, including *Ericaria zosteroides* (Reynes, Aurelle, et al. 2021), the *Ericaria selaginoides* complex (Bermejo et al. 2018), and *Gongolaria barbata* (Riquet et al. 2021), all of which exhibit strong population structuring despite varying degrees of oceanographic connectivity. Collectively, these studies indicate that restricted effective dispersal and limited gene flow are common features of Mediterranean canopy‐forming Fucales and represent major drivers of their population genetic structure. A comparable pattern has also been observed in other Mediterranean brown algae outside the *Cystoseira* complex, such as the kelp *Laminaria rodriguezii*, where strong genetic differentiation among populations has similarly been linked to limited dispersal and habitat fragmentation (Reynes, Thibaut, et al. 2021).

To further investigate the role of geographic isolation, AMOVA was also conducted comparing mainland and island populations, revealing even more pronounced structuring (Φ_CT_ = 0.64917; Φ_SC_ = 0.60393; Φ_ST_ = 0.86105; *p* < 0.05). Most of the genetic variation (64.91%) is due to differentiation between the two groups, highlighting the major role of geographic isolation of islands in genetic divergence. Island environments, often characterized by specific ecological conditions and small population sizes, favor genetic drift and significantly reduce gene flow with continental populations (Neiva et al. 2012; Bermejo et al. 2018). This pattern can be explained by the combined effects of geographic isolation and limited dispersal across fragmented coastal habitats in the Mediterranean, where habitat discontinuity and oceanographic transport interact to shape stepping‐stone connectivity and promote genetic differentiation (Buonomo et al. 2017). In Fucales, dispersal is highly restricted because zygotes rapidly sink and recruitment occurs close to parental individuals, leading to strong spatial genetic structure (Mangialajo et al. 2012). Consequently, even short geographic distances can result in strong genetic breaks when suitable rocky habitats are discontinuous and dispersal success is low (Coleman and Brawley 2005; Thibaut et al. 2016). Over time, reduced gene flow and genetic drift in small, isolated populations lead to progressive divergence between island and mainland groups, resulting in the strong genetic structure observed in this study (Wright 1951).

Taken together, all the AMOVA results confirm that *E. amentacea* populations present a complex and hierarchical genetic structure, dominated by allopatric differentiation processes (mainland/island), but partially attenuated by regional connectivity among geographically close populations. This population structure is supported by network analysis and phylogenetics. The specimens from the Ligurian region (Pontetto, Bergeggi, and Bonassola) formed a well‐supported monophyletic group, indicating high genetic homogeneity among northern populations and suggesting continuous gene flow along the Ligurian coastline. Within this cluster, minor sub‐structuring was observed among sites, which may reflect local adaptation or limited dispersal between nearby coastal habitats. Gene flow analysis confirms these results. The highest values of Nm are observed between Pontetto, Bergeggi, and Bonassola (Nm > 4). A comparable but contrasting pattern has been reported by Medrano et al. (2020) for *Treptacantha elegans* ((C. Agardh) De Clerck) along the Catalan coast, where populations showed high genetic homogeneity and weak differentiation despite spatial separation. In that system, connectivity was facilitated by coastal currents and the dispersal of drifting fertile fragments, with the Medes Islands acting as a potential source population for regional expansion. However, unlike *T. elegans* , where these mechanisms appear sufficient to maintain regional connectivity, our results suggest that similar dispersal processes are not enough to overcome the combined effects of habitat discontinuity and geographic isolation in *E. amentacea*, resulting in much stronger population structuring.

The distinct clustering of *E. amentacea s*icilian samples (Capo Milazzo) together with those from Apulia into a separate lineage, supported by moderate bootstrap values (65%–52%), reflects a clear genetic differentiation likely driven by geographical isolation and the biogeographic role of the Strait of Messina as a semi‐permeable barrier to gene flow. This pattern is consistent with previous phylogeographic studies on Mediterranean marine taxa showing that the Strait of Messina, separating the Tyrrhenian and Ionian basins, represents a major genetic discontinuity zone due to its complex hydrodynamics, strong currents, and contrasting ecological conditions. In *E. amentacea*, microsatellite data revealed strong structuring along southern Italy driven by habitat discontinuity and stepping‐stone oceanographic dispersal, with gene flow influenced by coastal configuration and current‐mediated connectivity (Buonomo et al. 2017). Similarly, *Posidonia oceanica* SSR analyses identified a displaced east–west genetic break shifted toward southern Calabria, with Strait of Sicily populations showing greater affinity with western lineages (Serra et al. 2010). Comparable evidence from marine invertebrates using COI markers further supports the Tyrrhenian–Ionian sector as a persistent phylogeographic barrier shaped by both historical isolation and contemporary oceanographic processes (Villamor et al. 2014; Sanna et al. 2013). Together, these studies suggest that the observed Sicilian–Apulian differentiation reflects the interaction of historical vicariance and present‐day semi‐permeable connectivity across the central Mediterranean.

Unlike the Sicilian–Apulian lineage, whose differentiation is primarily associated with central Mediterranean biogeographic barriers, the genetic structure of Sardinian populations may reflect the long‐term geological evolution of the western Mediterranean. The Oligocene–Miocene counterclockwise rotation of the Corsica–Sardinia microplate and the opening of the Liguro‐Provençal Basin progressively isolated Sardinia from the Ligurian region and the northwestern Mediterranean. Subsequently, the opening of the Tyrrhenian Sea and the formation of the Apennine chain reshaped the Italian peninsula, increasing the geographic separation between northern and southern Mediterranean regions (Advokaat et al. 2014; Schmitt et al. 2021). These tectonic events established the current configuration of the Mediterranean, providing the geological framework upon which later events, including the Messinian Salinity Crisis, Quaternary sea‐level fluctuations, and contemporary oceanographic circulation, likely contributed to the deep genetic divergence observed in Sardinian *E. amentacea* populations relative to mainland lineages. Within Sardinia, populations separated into two geographically coherent clusters: one containing samples from Corona Niedda and the other from Torre dei Corsari. This genetic distinction between northern and southern Sardinian sites (bootstrap 73%) indicates low connectivity and likely regional divergence across the island, which could be caused by hydrodynamic patterns or habitat discontinuities that affect propagule dispersal (Buonomo et al. 2017; Piazzi et al. 2018). The Pantelleria samples, which represent a separate and very different clade at the base of the *E. amentacea* group, had the highest genetic divergence from all other populations. This pattern provides evidence that the Pantelleria population is strongly isolated, which is quite expected due to its marine island position in the Strait of Sicily and the consequent long‐term isolation from mainland and island populations. The gene flow and Fst analyses support this finding: Nm values range from 0 to 0.06, while Fst ranges from 0.081 to 1 in Pantelleria and other Italian regions.

The concordance between the Neighbor‐Joining phylogeny and the haplotype network and Molecular variance analysis strongly supports the existence of geographically structured lineages within *E. amentacea* across the Italian coasts. Both analyses revealed a clear separation between all Italian‐sampled coast sites as well as Islands‐mainland Italian populations, with Ligurian samples forming a cohesive genetic group characterized by high haplotype sharing and low mutational divergence, consistent with continuous connectivity along the northern mainland.

The phylogeographic structure observed in *E. amentacea* populations has important implications for the conservation and management of these threatened brown algae. The observed genetic differentiation among regional populations, particularly between the Ligurian, Sicilian, Sardinian, and Pantelleria groups, suggests limited linkage and gene flow throughout the species' range. Such genetic variety implies that each regional population may be a potentially evolutionarily significant unit (ESU) that contributes to the species' adaptive capacity. Certain populations, such as Pantelleria and southern Sardinia, are particularly vulnerable to local extinction since recolonization through migration from other locations is unexpected. This pattern of significant population structure suggests that conservation efforts should use a geographically varied approach rather than a uniform management plan. These findings support the idea that effective conservation of canopy‐forming brown algae necessitates the incorporation of phylogeographic data into conservation marine reserve design and Maritime Spatial Planning policies, ensuring that management networks include the entire range of genetic variation across the species' distribution.

## 5 Conclusion

These phylogeographic patterns have important implications for conservation. The marked genetic divergence among regions, especially between Ligurian, Sicilian, Sardinian, and Pantelleria populations, suggests that each may represent an evolutionarily significant unit (ESU) with unique adaptive potential. Populations in Pantelleria and southern Sardinia may be particularly vulnerable to local extinction, as recolonization from other regions appears unlikely. Consequently, conservation strategies should adopt a region‐specific approach rather than uniform management across the species' range. Integrating phylogeographic data into marine spatial planning and reserve design is therefore essential for effective conservation of canopy‐forming brown algae. Protecting a network of sites that captures the full spectrum of genetic diversity will help ensure the long‐term resilience of the Mediterranean *E. amentacea* and the coastal ecosystems it supports.

## Author Contributions

**Maha Moussa:** conceptualization (lead), data curation (lead), formal analysis (lead), methodology (lead), software (lead). **Sarra Choulak:** formal analysis (lead), methodology (lead), software (lead), writing – original draft (equal), writing – review and editing (lead). **Valentina Asnaghi:** methodology (equal), writing – review and editing (equal). **Daniele Grech:** methodology (equal), writing – review and editing (equal). **Gareth Anthony Pearson:** methodology (equal), software (supporting). **Khaled Said:** methodology (equal), visualization (lead), writing – review and editing (lead). **Mariachiara Chiantore:** project administration (lead), supervision (lead), validation (lead), visualization (lead), writing – review and editing (lead). **Sonia Scarfi:** project administration (lead), supervision (lead), validation (lead), visualization (lead), writing – review and editing (lead).

## Funding

This study was funded by the project “National Biodiversity Future Center—NBFC,” funded under the National Recovery and Resilience Plan (NRRP), Mission 4 Component 2 Investment1.4—Call for tender No. 3138 of 16 December 2021, rectified by Decree No. 3175 of 18 December 2021 of the Italian Ministry of University and Research, funded by the European Union—NextGenerationEU; Award Number: Project code CN_00000033, Concession Decree No. 1034 of 17 June 2022, adopted by the Italian Ministry of University and Research, CUP D33C22000960007.

## Conflicts of Interest

The authors declare no conflicts of interest.

## Data Availability

COI DNA sequences: Genbank accessions PX906911–PX906952.
